# Oxygen Radical-Generating Metabolites Secreted by Eutypa and Esca Fungal Consortia: Understanding the Mechanisms Behind Grapevine Wood Deterioration and Pathogenesis

**DOI:** 10.3389/fpls.2022.921961

**Published:** 2022-07-04

**Authors:** Gabriel Perez-Gonzalez, Dana Sebestyen, Elsa Petit, Jody Jellison, Laura Mugnai, Eric Gelhaye, Norman Lee, Sibylle Farine, Christophe Bertsch, Barry Goodell

**Affiliations:** ^1^Department of Microbiology, University of Massachusetts, Amherst, MA, United States; ^2^Stockbridge School of Agriculture, University of Massachusetts, Amherst, MA, United States; ^3^Center for Agriculture, Food and the Environment, University of Massachusetts, Amherst, MA, United States; ^4^Department of Agricultural, Food, Environmental and Forestry Science and Technology, University of Florence, Firenze, Italy; ^5^INRAE, IAM, Université de Lorraine, Nancy, France; ^6^Chemical Instrumentation Center (CIC), Boston University, Boston, MA, United States; ^7^Laboratoire Vigne Biotechnologies et Environnement, Université de Haute-Alsace, Colmar, France

**Keywords:** grapevine wood, ascomycota fungi, chelator-mediated fenton (CMF) catalysis, fungi, wood decay, hydroxyl radical (OH^•^), cellulose, lignin

## Abstract

Eutypa dieback and Esca complex are fungal diseases of grape that cause large economic losses in vineyards. These diseases require, or are enhanced by, fungal consortia growth which leads to the deterioration of the wood tissue in the grapevine trunk; however, pathogenesis and the underlying mechanisms involved in the woody tissue degradation are not understood. We examined the role that the consortia fungal metabolome have in generating oxygen radicals that could potentially play a role in trunk decay and pathogenesis. Unique metabolites were isolated from the consortia fungi with some metabolites preferentially reducing iron whereas others were involved in redox cycling to generate hydrogen peroxide. Metabolite suites with different functions were produced when fungi were grown separately vs. when grown in consortia. Chelator-mediated Fenton (CMF) chemistry promoted by metabolites from these fungi allowed for the generation of highly reactive hydroxyl radicals. We hypothesize that this mechanism may be involved in pathogenicity in grapevine tissue as a causal mechanism associated with trunk wood deterioration/necrosis in these two diseases of grape.

## Introduction

**FIGURE 1:**
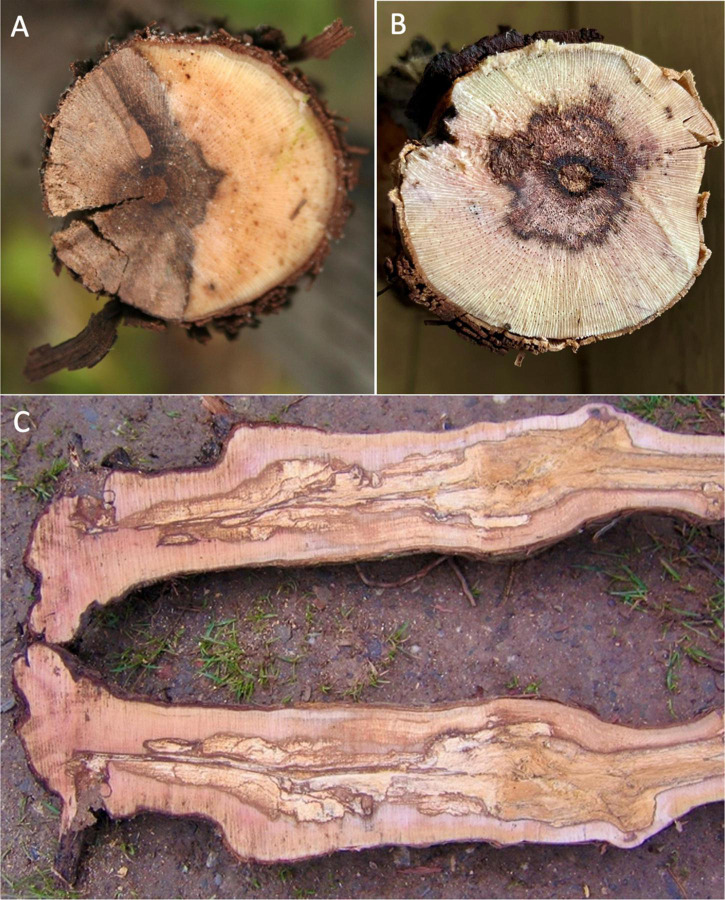
**(A)** Cross-section of grapevine trunk with necrosis and wood degradation typical of Eutypa dieback. **(B,C)** Cross-section and longitudinal sections, respectively, of grapevine trunk with necrosis and wood degradation typical of Esca disease.

Grapevine trunk diseases (GTDs) are caused by various fungi, with some GTDs being associated with multiple or a complex of fungi. Increasing damage to vineyards by GTD fungi has been noted since the end of the 20th century, with most GTD fungi attacking the perennial woody tissues of the stem, ultimately leading to the death of the grapevine (Surico et al., 2006; Bertsch et al., 2009; Bertsch et al., 2013). GTDs are characterized by the dieback and necrosis/decay of the woody tissue of the vine (Figure 1). Some of these diseases can show foliar symptoms that may not appear until deterioration of the stem wood is advanced (Labois et al., 2020). Although Eutypa dieback has been reported to be caused solely by *Eutypa lata* or other *Eutypa* species, current literature suggests that other pathogens can also be associated with Euypa dieback. Furthermore, *Eutypa* spp. is often associated with a consortium of other fungi, especially the Diaporthales order, with *Phaeoacremonium minimum* (Pmin) and *Phaeomoniella chlamydospora* (Pch) predominating (Rolshausen et al., 2013; Mondello et al., 2018). Eutypa dieback, Esca, and *Botryosphaeria* dieback are the most significant GTDs involving one or several xylem-inhabiting fungi, with Pch and Pmin typically found in consortia for Esca disease development (Bertsch et al., 2013; Bruez et al., 2013).

Most GTD fungal pathogens enter grapevine trunk wood in vineyards through pruning wounds, inhabiting the xylem cells in the woody tissue and causing, with time, significant necrosis, and decay, ultimately leading to foliar symptoms and cordon and vine death (Labois et al., 2020). In Eutypa dieback, as the decay and disease progresses, complete loss of yield, stunting of shoots, and/or loss of cordons and vines occur, with older vineyards experiencing as much as 30% necrosis of cordons or vines. In the United States, Eutypa dieback and *Botryosphaeria* dieback predominate in California and have also been an emerging issue for cold-climate vineyards in the Northeastern US and in British Colombia, Canada. In California alone, losses by GTDs each year amount to 14% of the value of the wine grapes produced with economic losses of more than $260 million per year (Siebert, 2001; Fontaine et al., 2016). GTDs also cause in the excess of US $1.5 billion in losses to vineyards globally each year (Hofstetter et al., 2012).

While pathogenic mechanisms and foliar damage in Eutypa dieback have been reported to be associated with the production of eutypine and other phytotoxic compounds (Tey-Rulh et al., 1991), the mechanisms involved in producing woody tissue degradation and other symptoms associated with both Eutypa dieback and Esca are still not well understood. Importantly, it is also unknown why a consortium of fungi is typically involved in both diseases (Tey-Rulh et al., 1991; Claverie et al., 2020).

Wood decay by brown rot Basidiomycota species is similar in some respects to the necrosis in grapevine wood caused by Ascomycota GTD fungi. Decay initiated by brown rot fungi produces a type of wood degradation where both holocellulose and lignin are depolymerized by a highly oxidative, non-enzymatic “chelator-mediated Fenton” (CMF) mechanism (Xu and Goodell, 2001; Arantes et al., 2012, Goodell, 2020; Hess et al., 2020). In brown rot, after, or concurrent with CMF action, the polysaccharide component of the wood cells is also selectively acted upon by fungal extracellular carbohydrate active enzymes (CAZymes). The lignin component is depolymerized by CMF action and then rapidly repolymerized within the wood cell wall (Goodell et al., 2017; Goodell, 2020). In *E. lata*-infected grape wood, similarly in some ways to brown rot wood decay, glucose and xylose from wood hemicelluloses are preferentially degraded and depleted from the wood whereas lignin is degraded much more slowly (Rolshausen et al., 2008). The residual lignin in both brown rotted wood and in necrotic wood attacked by GTD fungi is brown in coloration because of the oxidized residual or modified lignin which remains. Because of the similarities to brown rot wood decay, and prior reports on the action of oxygen radical generation in Esca disease by fungal metabolites (Osti and Di Marco, 2010), we considered that a mechanism similar to CMF chemistry might potentially play a role in GTD fungal attack of grapevine wood. The hallmark of the CMF system is the production of fungal low molecular weight (LMW) compounds that promote the CMF reaction which ultimately results in Fenton chemistry occurring (equation (1)) where Fe^3+^ is reduced to Fe^2+^ within the wood cell wall, and away from the fungal hyphae (Xu and Goodell, 2001; Goodell, 2020). CMF chemistry is promoted by a reduction in pH of the fungal microenvironment. Brown rot fungi generally reduce the pH of their microenvironments often lower than pH 4, and this is thought to aid in promoting a sequence of reactions leading to the Fenton reaction occurring within the higher pH wood cell wall (Goodell et al., 1997; Contreras et al., 2007; Goodell, 2020). In the low pH environment of the brown rot fungal extracellular matrix (ECM) which immediately surrounds the fungal hyphae, the pH is lower than pH 4.0 and iron will be sequestered (Goodell, 2020). However, within the wood cell wall, the pH is maintained at approximately 5.5, and at that pH, LMW compounds will redox cycle with iron to generate Fe^2+^ and also generate H_2_O_2_ (Akagawa et al., 2003; Varela and Tien, 2003). Both reactants are required for the generation of hydroxyl radicals (HO^•^), which must be generated within the cell wall for oxidative depolymerization of both cellulose and lignin to occur. This action generates diffusible LMW oligosaccharides from the cell wall which can then be acted upon by fungal CAZymes (Goodell et al., 2017; Zhu et al., 2020).


(1) $$\text{F}{\text{e}}^{2+}+{\text{H}}_{2}{\text{O}}_{2}\overset{\text{pH}3-5.5}{\to }\text{F}{\text{e}}^{3+}+\text{O}{\text{H}}^{-}+\text{O}{\text{H}}^{.}$$

Osti and DiMarco reported that fungal supernatants of Pch and Pmin contained phenolate siderophore-like compounds, and their analysis suggested that catecholate compounds from the fungal supernatants were present that could also reduce iron, generate hydroxyl radicals (HO^•^), and depolymerize cellulose (Osti and Di Marco, 2010). However, their supernatants also contained high molecular weight components such as extracellular enzymes which could have skewed their results. *Eutypa lata* (Elata) and other fungal species involved in GTDs are known to produce acetylenic phenols and heterocyclic analogs similar to some types of siderophores (Molyneux et al., 2002; Mahoney et al., 2003), and several LMW phenolic metabolites have been identified from Pmin and Pch (Andolfi et al., 2011). It has not, however, been reported whether these compounds have the ability to redox cycle or reduce iron as has been observed with some types of catecholate compounds produced by brown rot decay fungi, such as 2,5-dimethoxyhydroquinone (Xu and Goodell, 2001).

Because of the limited information about the role of LMW fungal metabolites related to GTD pathogenicity, we examined the metabolites from individual Elata, Pmin, and Pch cultures and also consortia-grown metabolites in our current work. We looked specifically at iron reduction and hydroxyl radical generation to determine whether there was a link between the LMW metabolites produced, and non-enzymatic chemistries that may generate hydroxyl radicals in a manner similar to that which has been demonstrated *via* CMF chemistry in Basidiomycota wood degrading fungi. Our objective was to examine whether chemistries similar to the CMF mechanism, promoted by LMW fungal metabolites, could potentially be generated by the GTD fungi when grown in culture. We did not assess for fungal siderophore activity in sequestering iron for fungi or plants, as the examination of classic siderophore function was not part of our objectives for this research.

## Materials and Methods

A total of three consortia fungi involved in Eutypa dieback or esca disease were assessed in this research: *Phaeomoniella chlamydospora* (Pch, isolate UCD7872), *Phaeoacremonium minimum* (Pmin, isolate UCD7770), and *Eutypa lata* (Elata, isolate UCD7746); all isolated from vineyards in Lodi, California. These fungi were grown both in single culture and in combination (consortia culture) (Elata_Pch; Elata_Pmin; Pmin_Pch) for 6 weeks in low-iron media as detailed below. LMW iron-binding metabolite production was assessed, as was hydroxyl radical generation. All analyses were conducted in triplicate unless otherwise stated.

### Culture Media

Iron-free cultures with restricted nutrient media were used to promote biosynthesis of iron-binding LMW compounds (Jellison et al., 1991), with all glassware acid washed in 10% HCl for 24 h, rinsed with deionized distilled water (ddH_2_O – 18.2MΩ.cm) followed by a 90 mM EDTA wash, and then rinsed 3 times with ddH_2_O. Restricted nutrient iron-free media, modified from Highley (1973), were used as follows: 1L of ddH_2_O mixed with 2 g ammonium nitrate (Sigma-Aldrich, MO, United States), 2 g monobasic potassium phosphate (Merck, MA, United States), 0.5 g magnesium sulphate heptahydrate (Sigma-Aldrich, MO, United States), 0.1 g calcium chloride (Bio Basics Canada Inc., ON, Canada), 0.57 mg boric acid (Sigma-Aldrich, MO, United States), 0.31 mg zinc sulphate heptahydrate (HIMEDIA labs, PA, United States), 0.039 mg copper sulphate pentahydrate (Acros Organic, Belgium), 0.036 mg manganese chloride tetrahydrate (Fisher Chemical), 0.018 mg ammonium molybdate tetrahydrate (Acros Organic, Belgium), and 0.001 g thiamine HCl (Acros Organic, Belgium). For carbon sources, glucose (Alpha Biosciences) 0.5% (w/v) and 50 μm microcrystalline cellulose (Acros Organics, Belgium) 1% (w/v) were used with five replicates for each culture and in each medium. The media solution (200 ml per 0.5-L flask) was brought to a pH of 5.5 using NaOH and autoclaved for 30 min. Liquid cultures were inoculated using mycelial slurries prepared by scraping mycelium from fully grown agar plates into 50 ml of sterile ddH_2_O. About 1 ml of mycelial slurry was carefully pipetted onto the surface of the liquid media in each flask, and cultures were incubated without shaking to promote production of LMW metabolites.

### Low Molecular Weight Metabolite Extraction

After 6 weeks of fungal growth, liquid cultures were coarsely filtered (Whatman #4 cellulose filters) to remove mycelium and cellulose microcrystals from the liquid cultures, followed by preliminary filtration through 0.22-μm cellulose filters under vacuum. Filtrates were then ultrafiltered through 5 kDa polyethersulfone filters (Amicon Stirred Cell filtration - EMD Millipore, MA, United States) to yield the <5 kDa LMW metabolite fraction. A Bradford assay confirmed that proteins were not present (results not shown). The LMW metabolite fraction was acidified to pH 3 with HCl prior to a triple ethyl acetate (1:1 with ultrafiltered fractions) extraction for phenolics (Jellison et al., 1991). The organic fraction was then dried under reduced pressure, resuspended in methanol, and filtered through a 0.22-μm filter to yield the final <5 kDa LMW extract.

### Determination of Total Phenols by the Folin-Ciocalteu Assay

Folin–Ciocalteu (FC) reagent was used to spectrophotometrically quantify (765 nm) the amount of total phenols in solution (Singleton et al., 1999) with gallic acid used as the standard. Samples of purified extract (20 μl) and the FC reagent (100 μl) were reacted (5 min @RT) before the addition of 20% sodium carbonate (300 μl) to initiate the FC reaction. After a further reaction period (2h @RT) in the dark, absorbance values were compared to the gallic acid standard for phenolic concentration determination.

### Determination of Iron Reduction by Ferrozine Assay

The Ferrozine assay is a colorimetric assay that is sensitive specifically to the ferrous form of iron, and it is therefore useful in measuring iron reduction capacity (Pierson and Clark, 1984). The final concentration of components in each reaction cuvette was: acetate buffer (0.100 mM, pH 5.5), Ferrozine (0.250 mM), and Fe^3+^ (0.030 mM). Phenolics in the fungal extract (15 mM according to FC assay) were added last to start the reaction. Ferrous chloride (FeCl_2_) was used for the standard. Samples were mixed thoroughly in each cuvette and the reaction followed spectrophotometrically (562 nm) at 5-min intervals for 45 min. The ferric iron reductive capacity of all LMW extracts was normalized per nmol of phenolics added to the reaction and reported as the amount of iron reduced (mol)/amount of phenolic (mol) in the extracts assayed.

### FOX Assay for H_2_O_2_ Detection

A ferrous ammonium – xylenol orange (FOX) assay (Wolff, 1994) was used to measure H_2_O_2_ evolution which occurs during the oxidation of ferrous iron in Fenton chemistry. The oxidized iron reacts with xylenol orange (XO) to form a complex detectable at 560 nm (Wolff, 1994; Rhee et al., 2010). MES buffer (50 mM, 5.5pH) and fungal extracts containing phenolics (15 mM according to FC assay) were added to 100 μl of the FOX reagent (a 1:1 ratio of the XO/Ferrous ammonium sulfate mixture and sorbitol) to yield a solution of 0.100 mM XO, 0.250 mM ferrous ammonium sulfate, 25 mM H_2_SO_4_, and 100 mM sorbitol, which was incubated (RT, 30 min) before centrifugation (12,000 g, 5 min) to remove any precipitate. Reactions were carried out under low light conditions to reduce UV interference, analyzed at 560 nm for H_2_O_2_ detection, and reported as the amount of H_2_O_2_ generated (mmol)/amount of phenolic (mol) in the extracts assayed.

### EPR for Detection of Hydroxyl Radicals

Methanol was evaporated from ultrafiltered samples using a SpinVac (35°C, 45 min), and the samples then resuspended in acetate buffer adjusted to pH 3.5 with HNO_3_. For hydroxyl radical detection in EPR, a DMPO spin-trap (5,5-dimethyl-1-pyrroline-n-oxide, 10 mM) was mixed with H_2_O_2_ (0.15mM) and a fungal extract (50mM adjusted per the FC assay). Fe^3+^ (0.15mM) was then added to start the reaction. (Final reactant concentrations are listed in all cases). After incubation (5 min, @RT), the samples were transferred to a 50 μl EPR capillary glass tube. Catechol (50 mM) was used as reference compound (Tamaru et al., 2019). Analysis was conducted in the X-band frequency (9.8 GHz) using a Bruker Elexsys-500 EPR instrument equipped with a super high QE cavity (ER4122SHQE-W1).

### High-Performance Liquid Chromatography (HPLC)

Low molecular weight extracts were analyzed using a Shimadzu HPLC system with an analytical C18 Nucleosil column (250 mm × 4.6 mm × 5 μm, 0.50 ml/min). Flow-through UV analysis (280 nm) was conducted using an acetonitrile (ACN) linear gradient (10–90%) over 45 min. The gradient was then held at 90% for 5 min and the gradient reversed over the next 5 min before completion with 10% ACN over a total of 60 min. Fractions were then scanned (190 370 nm) to detect potential phenolic compounds at ∼280 nm absorption.

### Identification of Metabolites by Mass Spectroscopy (UPLC-MS)

Fungal extracts were analyzed by liquid chromatography including screening with electrospray ionization MS (Ultimate 3000 LC with an ACQUITY HSS T3 100 mm × 2.1 mm × 1.8 μm UPLC column combined with a Thermo Q Exactive ESI-MS). The mobile phase was as follows: solvent A (0.05% formic acid water) and solvent B (acetonitrile) with a gradient elution (0-1.0 min, 5%B; 1.0–12.0min, 5–95%B; 12.0–13.5min, 95%B; 13.5–13.6 min, 95–5%B; 13.6–16.0 min, 5%B). The mobile phase flow rate = 0.3 ml⋅min^–1^. Column temperature (40°C) and the sample manager (4°C) were both constant.

Mass spectrometry parameters in ESI + and ESI- modes were as follows:

**ESI +** : Heater Temp 300°C; Sheath Gas Flow rate, 45arb; Aux Gas Flow Rate, 15arb; Sweep Gas Flow Rate, 1arb; spray voltage, 3.0KV; Capillary Temp, 350°C; S-Lens RF Level, 30%.

**ESI-**: Heater Temp 300°C, Sheath Gas Flow rate, 45arb; Aux Gas Flow Rate, 15arb; Sweep Gas Flow Rate, 1arb; spray voltage, 3.2KV; Capillary Temp,350°C; S-Lens RF Level,60%.

Compounds were identified using Compound Discoverer™ (ThermoFisher Scientific) a high-resolution accurate-mass data software for metabolite identification. This software does not contain a database, but it is a software tool that compares the experimental MS data to available databases such as ChemSpider, mzVault, mzCloud, KEGG, and other smaller databases for the identification of unknown.

## Results

Overall, when cultures of two fungi were grown in consortia in some instances, the cultural appearance of one of the species was maintained. This always occurred when Pmin was part of the consortia. With Elata_Pch consortia growth, discrete colonies of both species were apparent in about half the cultures whereas in the other half, Elata growth predominated. However, when comparing the metabolomic profile of the individual cultures to the consortia growth, unique extract profiles were observed in the consortia cultures as detailed below and in Supplementary Figure 1.

### Production of Phenolics and Iron Reduction Capacity

**FIGURE 2:**
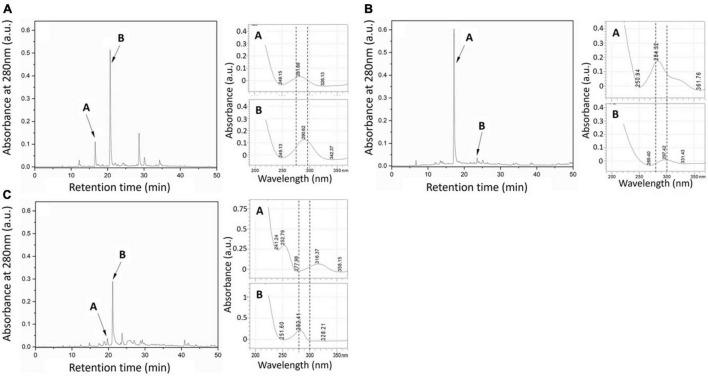
HPLC chromatograms (left for each sample) of **(A)** Elata, **(B)** Pmin, and **(C)** Pch fungal metabolite extracts taken at 280 nm. Labels (A,B) indicate the putative phenolic peaks selected (abs. ∼280 nm). (Right) UV spectra of the two most abundant extract peaks.

Preliminary chromatographic screening of all fungal extracts by HPLC for putative phenolic compounds (280 nm) showed that in all cultures, a maximum of five detectable peaks was produced (Figures 2A–C). The two most prominent phenolic peaks for extracts from the three fungi are shown in Figure 2.

The Folin–Ciocalteu (FC) assay for phenolics in individual ∼280 nm UV fractions showed that Pch extracts contained the greatest amount of phenolics when compared to either the Elata or Pmin individual fungal culture extracts (Table 1, *p* < 0.001, *p* < 0.001). Extracts from all fungal consortia yielded an increase in total phenolics over individual cultures (Table 1). The Elata_Pmin consortia produced significantly more phenolics than either Elata or Pmin alone (Table 1, *p* = 0.01, *p* = 0.03). For the Elata_Pch consortia growth, there was an averaging effect relative to phenolic production. Phenolic content when Elata and Pch were grown in combination was significantly lower than Pch alone (*p* < 0.001), but it was significantly higher than Elata alone (*p* = 0.007). The Pmin_Pch combination showed an increase in overall phenolic production compared to that when the fungi were grown individually.

**TABLE 1:** Total phenolic content (Folin–Ciocalteu) of LMW fungal extracts from the three GTD fungi, together with iron reduction (Ferrozine), and H_2_O_2_ production (FOX) from those extracts.

	*Total phenolic content (*mM*)*	Iron reduced (mol Fe^2+^/mol of phenols)	H_2_O_2_ produced (mmol H_2_O_2_/per mol phenols)
Elata	1.03 ± 0.08	0.531 ± 0.010	690 ± 70
Pmin	1.07 ± 0.10	0.615 ± 0.013	800 ± 20
Pch	3.33 ± 0.36	1.34 ± 0.08	1.6 ± 0.1
Elata_Pmin	1.22 ± 0.107	0.877 ± 0.026	8.8 ± 0.3
Elata_Pch	2.25 ± 0.25	1.16 ± 0.05	1.7 ± 0.2
Pmin_Pch	4.05 ± 0.20	1.62 ± 0.10	0.4 ± 0.1

*Results shown as mean ± SD, n = 3.*

All culture extracts were found to be capable of reducing iron. Elata and Pmin extracts both reduced approximately the same level of iron per mol of phenols (0.531 and 0.615 mol iron/mol of phenolic, respectively). Pch extracts reduced more iron than the other two fungi per mol of phenolics (1.34 mol Fe^2+^/mol of phenolic) reflecting the greater phenolic content (from the FC results above). Pch extracts also displayed a greater level of reduction per mol of phenolic, reducing more than double the amount of iron compared to the extracts from Elata and Pmin in culture alone. The Elata_Pmin consortia extracts showed a significantly increased level of iron reduction when compared to each fungus grown alone (*p* < 0.001, *p* = 0.001), but the reduction level was not great enough to be considered an additive effect. The Pch_Pmin consortia extracts reduced less iron when compared to the extracts from each of the fungi when grown separately and added together (*p* = 0.035, *p* = 0.005). Iron reduction in the Elata_Pch consortia fungal cultures was significantly greater than from Elata alone (*p* = 0.003), and the reduction level was comparable to the Pch extracts when grown alone (*p* = 0.06).

### H_2_O_2_ Production

Extracts from Elata and Pmin produced the largest amounts of H_2_O_2_ (690 and 800 mmol H_2_O_2_/mol of phenols) as determined by the FOX assay (Table 1). While Pch and all combinations had detectable levels of H_2_O_2_ generation, they were in the 0.4 to 9 mmol range and significantly lower than both Elata and Pmin (Pch vs. Elata: *p* = 0.006, Pch vs. Pmin: *p* < 0.001, Elata vs. Pch_Elata: *p* = 0.006, Pmin vs. Pch_Pmin: *p* = 0.005).

### Electron Paramagnetic Resonance

**FIGURE 3:**
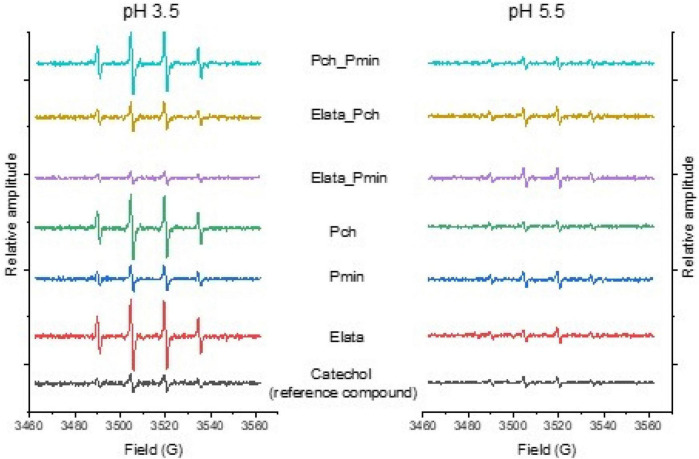
Electron paramagnetic resonance (EPR) spectra of GTD fungal extracts spiked with DMPO to detect hydroxyl radicals. The relative amplitude of each 4-peak spectra reflects the amount of hydroxyl radical produced relative to a catechol standard. Fungi were grown alone, and in consortia, to produce the extracts analyzed in this work.

The formation of a DMPO-HO^•^ adduct with its characteristic 4-line spectrum allowed detection and semi-quantification of HO^•^ in solution, with all extracts being found to generate HO^•^ in electron paramagnetic resonance (EPR) spin trapping at pH values of both 3.5 and 5.5 (Figure 3). The production of HO^•^ was considerably greater at pH 3.5 for most of the treatments tested. It should be noted that only Pch drove the culture medium to pH 3–3.5 under the experimental conditions tested. Uniquely, all extracts produced more HO^•^ than the reference catechol compound (catechols typically generate high HO^•^ levels under physiological conditions, alone or in the presence of iron (Miura et al., 1998; Forooshani et al., 2017)), when used at the same concentration based on FC analysis of phenolics.

### Identification of Low Molecular Weight Metabolites

Metabolite identification analysis of the three fungi in individual cultures (Supplementary Figures 2–4) showed that several phenolic and non-phenolic compounds were present, with some of them previously reported as having iron reduction capacity (Table 2). Interestingly, all fungi produced small catecholates including: 3,4-dihydroxybenzoic acid (Elata), caffeic acid (Pmin), and hydroxycinnamic acids such as sinapinic acid (Pch, Pmin) and caffeic acid (Pmin). In addition, important iron reducing metabolites identified in other fungal systems such as pyochelin (tetradentate coordination with iron) and terrain (specific coordination with iron unreported) were also detected in the Elata extract (Cox, 1986; Gressler et al., 2015; Asfour et al., 2019).

**TABLE 2:** Metabolites produced by *E. lata*, *P. minimum*, and *P. chlamydospora* with previously reported capacity for iron reduction.

Compound	Formula	Elata	Pmin	Pch	References
Pyochelin	C_14_H_16_N_2_O_3_S_2_	•			Coffman et al., 1990
3,4-dihydroxybenzoic acid	C_7_H_6_O_4_	•			Spiegel et al., 2020
Terrein	C_8_H_10_O_3_	•			Gressler et al., 2015
Sinapic acid methyl ester	C_12_H_14_O_5_		•	•	Siger et al., 2013
Dihydroferulic acid	C_10_H_12_O_4_			•	Chen et al., 2016
Caffeic acid	C_9_H_8_O_4_		•		Gülçin, 2006
Gallic acid	C_7_H_6_O_5_		•		Powell and Taylor, 1982
Homogentisic acid	C_8_H_8_O_4_		•		Martin and Batkoff, 1987
Sinapinic acid	C_11_H_12_O_5_		•		Hynes and O’Coinceanainn, 2004

Mass spectroscopy analysis also identified other organic phenols, aldehydes, and carboxylic acids (Table 3) without previously reported iron reduction activity; however, structures with multiple coordinating oxygens suggest the possibility for metal-ligand coordination with either bidentate or multidentate ligation. Further experiments with individual model metabolites would be required to demonstrate this with specific model compounds.

**TABLE 3:** Mass spectral analysis of phenolic, aldehydes, and carboxylic acid metabolites produced by *E. lata*, *P. minimum*, and *P. chlamydospora* without reported iron reduction activity.

Compound	Formula	Structure	Elata	Pmin	Pch
3,4,5-trimethoxycinnamic acid	C_12_H_14_O_5_	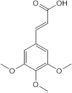	•		
Polygonolide	C_12_H_12_O_4_	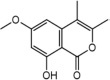	•		
3,4’,5-Biphenyltriol	C_12_H_10_O_3_	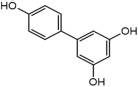	•		
4-hydroxy-3-(3-methylbut-2-enyl) benzoic acid	C_12_H_14_O_3_	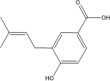		•	•
4,6,8-trihydroxy-7-methoxy-3-methyl-3,4-dihydroisochromen-1-one (Lignicol)	C_11_H_12_O_6_	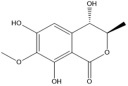		•	
3-coumaric acid	C_9_H_8_O_3_	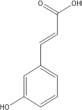		•	
Homovanillic acid	C_9_H_10_O_4_	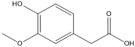			•
3-Methoxybenzaldehyde	C_8_H_8_O_2_	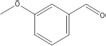			•
1-(3-ethyl-2,4-dihydroxy-6-methoxyphenyl)butan-1-one (Deoxyphomalone)	C_13_H_18_O_4_	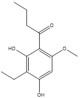			•

## Discussion

Our results demonstrate that all 3 GTD causative fungi, Elata, Pmin, and Pch, produced LMW phenolic metabolites, with greater amounts of total phenolics in many cases produced in consortia cultures. Although this may simply reflect a ramping up of defense phenolics in the presence of other microorganisms, it provides insight into alternative functions for those LMW phenolics because the iron redox and hydroxyl-generating functions will be activated at appropriate pH no matter the proposed role of those metabolites in the prior literature. We propose that the iron reduction and ROS-generating chemistries that we observed in this research could potentially be employed by fungi involved in Eutypa dieback and Esca complex diseases to promote woody tissue degradation in the grapevine trunk, and over time, even broader pathogenicity, such as foliar decline, within the grapevine host.

Mass spectroscopy identification confirmed the production of small catecholates including 3,4-dihydroxybenzoic acid (Elata) and caffeic acid (Pmin), and hydroxycinnamic acids such as sinapic acid methyl ester (Pch, Pmin) and dihydroferulic acid (Pch) (Table 2). These and similar compounds are known to enhance ROS production though CMF chemistry under appropriate conditions (Salgado et al., 2013). In addition, Elata also produced pyochelin, a phenolic siderophore, and iron reducing compound that is one of the primary siderophores isolated from *Pseudomonas* spp. (Brandel et al., 2012); and terrein, a non-phenolic iron reducing compound also produced by *Aspergillus terreus* with reported anticarcinogenic activity (Asfour et al., 2019).

Other LMW metabolites identified in the Elata, Pmin, and Pch cultures had structures that suggested iron chelation activity. However, further experiments with individual model metabolites would be required to demonstrate that activity with greater certainty.

As detailed in the CMF mechanism for brown rot fungi (Goodell et al., 1997; Goodell, 2020), H_2_O_2_ reacts with reduced iron in acidified environments (pH = 5.5) to promote HO^•^ and ROS generation within the cell wall to initiate oxidative deconstruction of the lignocellulosic components. Once degradation initiates, nanoscale pores in the wood cell wall are generated to promote diffusion of sugars and oligosaccharides out of the wood cell wall. Extracellular enzymes such as CAZymes can then act to digest oligosaccharides diffusing from the cell wall (Flournoy et al., 1991; Arantes et al., 2012; Cragg et al., 2015; Goodell et al., 2017).

Analyses for iron reduction and H_2_O_2_ generation by LMW extracts showed that both Elata and Pmin possessed relatively limited iron-reduction capacity, but had significantly greater capacity for production of H_2_O_2_ than Pch. However, only Pch-containing cultures reduced the pH of the media to the 3.0-3.5 range. Enzymes known to generate H_2_O_2_ are unable to penetrate intact plant cell walls (Tepfer and Taylor, 1981; Flournoy et al., 1991; Zhu et al., 2017) and because of the short half-life of HO^•^ radicals, their generation directly within the cell wall is necessary to depolymerize both cellulose and lignin. A LMW catalytic source for H_2_O_2_ within the cell wall would promote a more efficient CMF reaction. Redox cycling of iron-binding metabolites with oxygen in acidic pH environments can act as catalytic sources of H_2_O_2_ in solution, thus promoting the production of HO^•^ (Akagawa et al., 2003; Danilewicz, 2011).

The differential capacity of Pch, Elata, and Pmin extracts to promote either iron reduction and/or H_2_O_2_ production suggests that HO^•^ generation may be favored when multiple fungi are involved rather than a single species. Although each individual species has the potential to promote CMF activity in culture to a limited extent, consortia growth often generated greater amounts of HO^•^ radicals. As related to trunk wood deterioration/necrosis and pathogenicity in grapevine tissue, and the association of Pch and Pmin in Esca complex diseases and fungal associations promoting Eutypa dieback, as proposed, some GTD fungi would preferentially promote iron reduction, whereas others would play a greater role in H_2_O_2_ generation. The CMF non-enzymatic hypothesis could potentially explain why consortia fungal activity is required for these diseases, and it provides an alternative pathway for GTD pathogenesis where LMW metabolites promote oxidative damage *via* CMF chemistry rather than previously reported mechanisms related to direct metabolite toxicity or enzymatic oxidation.

One unexplained data variation, the extracted metabolites from the Elata_Pmin combination, produced H_2_O_2_ at a level of 10 mmol, but this was still well below the value for single cultures of Elata and Pmin, though still significantly higher than the Pch extract alone (*p* < 0.001). The basis for the reduced production of H_2_O_2_ is not known but may be due to the changes in the metabolite profile that occurs when the Elata_Pmin consortia are grown compared to the metabolites produced in individual culture (Supplementary Figure 1).

**FIGURE 4:**
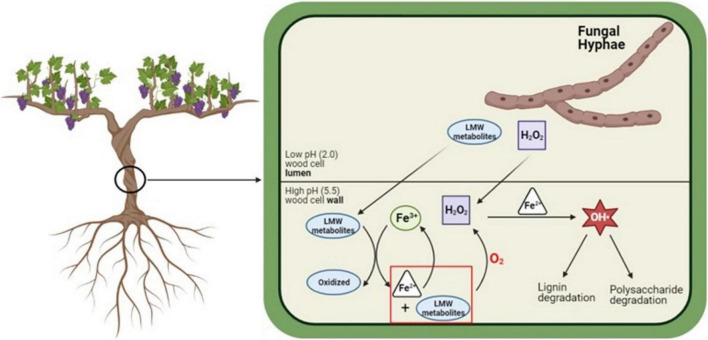
Proposed mechanism for the *in situ* generation of Fe^2+^ and H_2_O_2_, and degradation of lignin and cell wall macromolecules by GTD fungi. LMW metabolites and H_2_O_2_ diffuse into the cell wall, where the LMW metabolites sequester Fe^3+^ from the cell wall environment and reduce Fe^3+^ to Fe^2+^. Through a type of mediated CMF reaction, Fe^2+^ and H_2_O_2_ react and generate hydroxyl radicals (OH^•^). Images built using Biorender software. Schematic modified from Zhu et al. (2020).

We propose that individual GTD fungi produce LMW metabolites with specialized and differentiated functions, with some fungal species taking on greater roles relative to different steps in the CMF chemical mechanism such as the control of pH, reduction of iron, or the generation of H_2_O_2_. It is possible that these pathogenic species evolved with suites of LMW metabolites initially produced by the fungi as a mechanism of defense against other microorganisms, and later evolving to function in the specialized consortia oxidation roles described in our current work. The mechanism we propose showing the potential role of CMF chemistry in GTD pathogenesis (Figure 4) provides a basis for further exploration of fungal consortium as a hypothesis and may help to address why multiple fungal species are required to promote diseases in the Esca complex and Eutypa dieback. Further exploration of this mechanism could potentially open new alternatives to better understand these diseases and ultimately open paths to explore the targets for the development of treatments to prevent or limit yield loss in vineyards.

## Data Availability Statement

The original contributions presented in the study are included in the article/Supplementary Material, further inquiries can be directed to the corresponding author/s.

## Author Contributions

BG: conceptualization, validation, project administration, and funding acquisition. DS, GP-G, and BG: methodology. DS, GP-G, and NL: formal analysis. DS and GP-G: investigation and writing – original draft preparation. BG and EG: resources. DS, GP-G, EP, JJ, LM, CB, SF, EG, and BG: writing, reviewing, and editing. BG, DS, and GP-G: visualization. BG, GP-G, JJ, and EP: supervision. All authors contributed to the article and approved the submitted version.

## Author Disclaimer

The contents are solely the responsibility of the authors and do not necessarily represent the official views of the USDA or NIFA.

## Conflict of Interest

The authors declare that the research was conducted in the absence of any commercial or financial relationships that could be construed as a potential conflict of interest.

## Publisher’s Note

All claims expressed in this article are solely those of the authors and do not necessarily represent those of their affiliated organizations, or those of the publisher, the editors and the reviewers. Any product that may be evaluated in this article, or claim that may be made by its manufacturer, is not guaranteed or endorsed by the publisher.
