# Supplementary material for: Oxygen Radical-Generating Metabolites Secreted by Eutypa and Esca Fungal Consortia: Understanding the Mechanisms Behind Grapevine Wood Deterioration and Pathogenesis
Source: Front Plant Sci. 2022 Jul 4;13:921961. doi: 10.3389/fpls.2022.921961 (PMC9327790; doi:10.3389/fpls.2022.921961)
Supplement: Supplementary file 1 [file Data_Sheet_1.PDF]

SUPPLEMENTAL DATA FILE

Oxygen radical-generating metabolites secreted by eutypa and Esca fungal consortia

Gabriel Perez-Gonzalez[1], Dana Sebestyen[1], Elsa Petit[2], Jody Jellison[3] Laura Mugnai[4], Eric Gelhaye[5], Norman Lee[6], Sibylle Farine[7], Christophe Bertsch[7], Barry Goodell[1*]

[1]Department of Microbiology, University of Massachusetts, Amherst, MA 01003, U.S.A

[2] Stockbridge School of Agriculture, University of Massachusetts, Amherst, MA 01003, U.S.A

[3] Center for Agriculture, Food and the Environment, University of Massachusetts, Amherst, MA 01003 U.S.A.

[4]Department of Agricultural, Food, Environmental and Forestry Science and Technology, University of Florence, Firenze 50144, Italy

[5]Université de Lorraine, INRAE, Interactions Arbres-Microorganismes, F-54000 Nancy, France

[6]Chemical Instrumentation Center (CIC), Boston University, Boston, MA 02215, U.S.A

[7]Laboratoire Vigne Biotechnologies et Environnement EA-3991. Université de Haute-Alsace, Colmar 68008, France

[*]Corresponding author: Barry Goodell; Email: bgoodell@umass.edu

Appendix A. Supplementary data

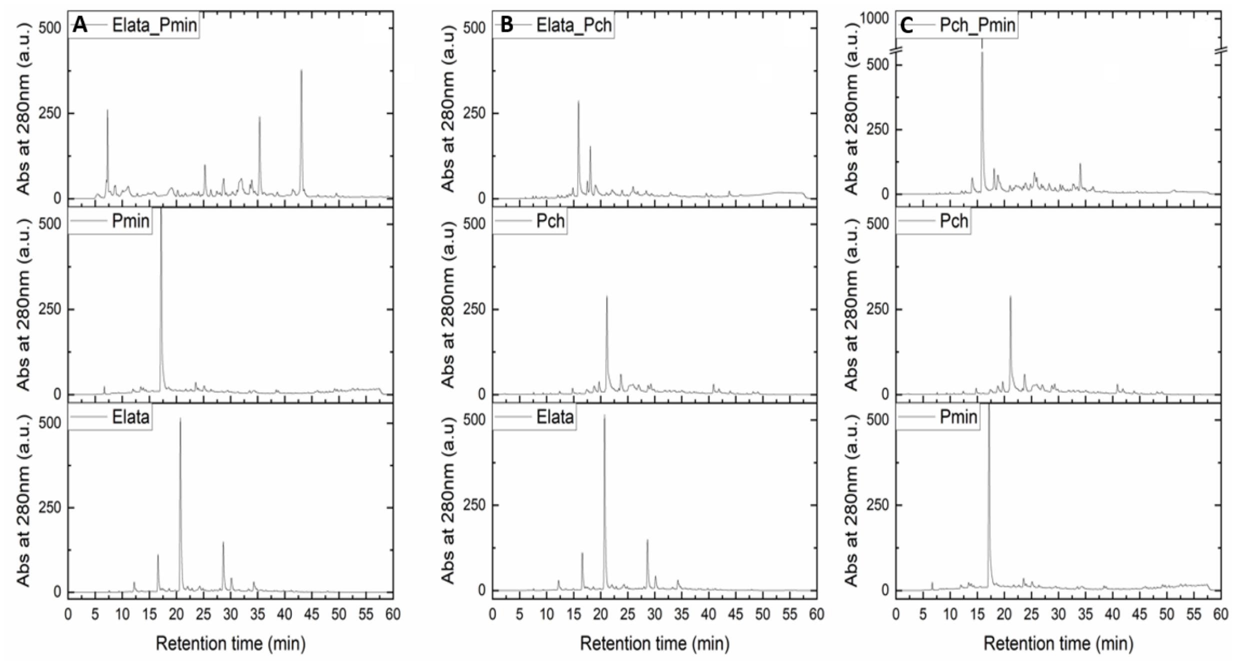

Fig. S1 HPLC chromatograms of Elata_Pmin (A), Elata_Pch (B) and Pch_Pmin (C) fungal metabolites with their respective single culture chromatograms. All absorbance values for metabolites were read at 280nm.

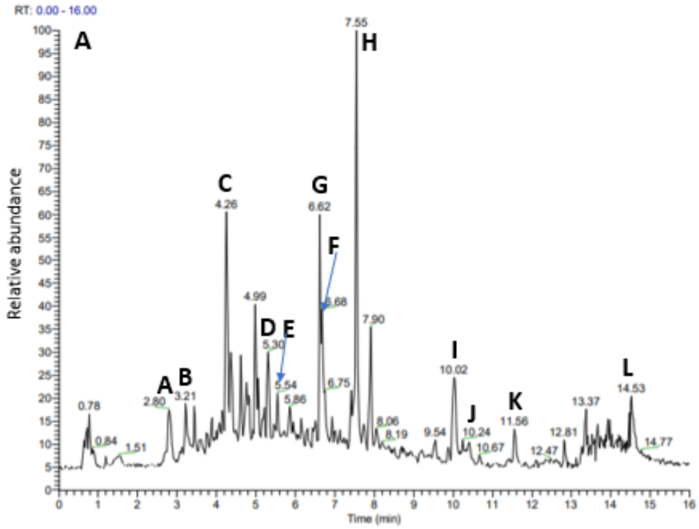

	Compound	Formula
A	Loganetin	$C_{11}H_{16}O_5$
B	Terrein[a]	$C_8H_{10}O_3$
C	Guaietolin	$C_{11}H_{16}O_4$
D	Polygonolide	$C_{12}H_{12}O_4$
E	Genipin	$C_{11}H_{14}O_5$
F	3,4',5-Biphenyltriol	$C_{12}H_{10}O_3$
G	Acetamiprid	$C_{10}H_{11}ClN_4$
H	Pyochelin[a]	$C_{14}H_{16}N_2O_3S_2$
I	Biocytin	$C_{16}H_{28}N_4O_4S$
J	Artemotil	$C_{17}H_{28}O_5$
K	Tretinoin	$C_{20}H_{28}O_2$
L	2,2',3,4',5,5',6-Heptachloro-4-biphenylol	$C_{12}H_3Cl_7O$

[a]Known iron reducer

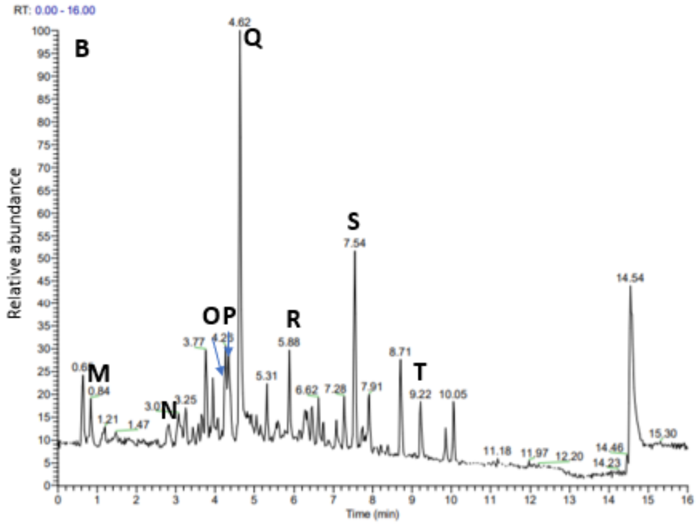

	Compound	Formula
M	2-Mercaptoethanol	C_2H_6OS
N	Dimethyl (3-oxocyclohexyl)malonate	$C_{11}H_{16}O_5$
O	Guaietolin	$C_{11}H_{16}O_4$
P	Streptol	$C_7H_{12}O_5$
Q	3,4-dihydroxybenzoic acid[a]	$C_7H_6O_4$
R	4-oxo-5-phenylpentanoic acid	$C_{11}H_{12}O_3$
S	Pyochelin[a]	$C_{14}H_{16}N_2O_3S_2$
T	2-(4-hydroxy-1,3-thiazol-2-yl)-1-phenylethan-1-one	$C_{11}H_{10}NO_2S$

[a]Known iron reducer

Fig. S2 Total Ion Current (TIC) chromatogram (left) and LMW phenolics produced (right) for *Eutypa lata* metabolite extracts in negative (A) and positive (B) ionization mode. Peaks were selected by largest area and peak height.

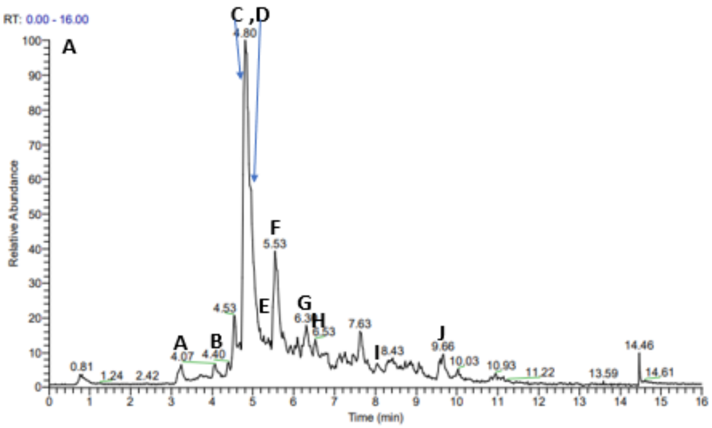

	Compound	Formula
A	Citrazinic acid	$C_6H_5NO_4$
B	7-Hydroxy-4-methylcoumarin	$C_{10}H_8O_3$
C	Sinapic acid methyl ester[a]	$C_{12}H_{14}O_5$
D	Dihydroferulic acid[a]	$C_{10}H_{12}O_4$
E	4-hydroxy-3-(3-methylbut-2-enyl)benzoic acid	$C_{13}H_{16}O_3$
F	Homovanillic acid	$C_9H_{10}O_4$
G	(2R)-7-hydroxy-8-(2-hydroxyethyl)-5-methoxy-2-methyl-2,3-dihydrochromen-4-one	$C_{13}H_{16}O_5$
H	2-[2-[(Z)-pent-2-enyl]-3-[3,4,5-trihydroxy-6-(hydroxymethyl)oxan-2-yl]oxycyclopentyl]acetic acid	$C_{18}H_{30}O_8$
I	Estrone-3-(beta-D-glucuronide)	$C_{24}H_{30}O_8$
J	Testosterone sulfate	$C_{19}H_{28}O_5S$

[a]Known iron reducer

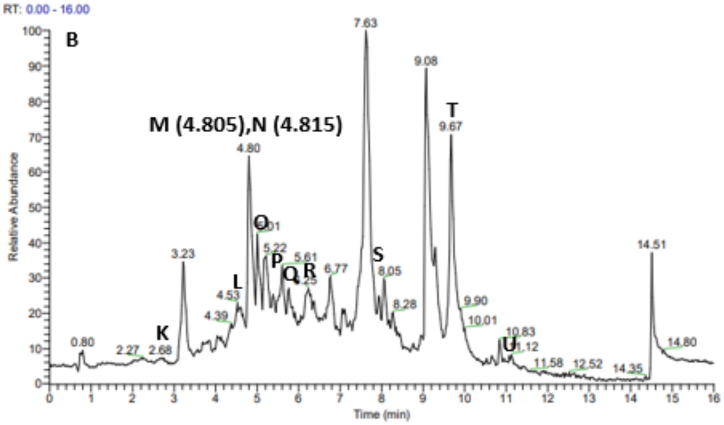

	Compound	Formula
K	Guvacoline	$C_7H_{11}NO_2$
L	L-(-)-Methionine	$C_5H_{11}NO_2S$
M	3-Methoxybenzaldehyde	$C_8H_8O_2$
N	1-(3-ethyl-2,4-dihydroxy-6-methoxyphenyl)butan-1-one	$C_{13}H_{18}O_4$
O	Jasmonic acid	$C_{12}H_{12}O_3$
P	Myristicin	$C_{11}H_{12}O_3$
Q	2-Hydroxybenzothiazole	C_7H_5NOS
R	Benzothiazole	C_7H_5NS
S	1-Dodecanoyllysolecithin	$C_{20}H_{42}NO_7P$
T	Eucalyptol	$C_{10}H_{18}O$
U	2-[1-(2H-1,3-benzodioxol-5-yl)propan-2-yl]-6-methoxy-4-(prop-2-en-1-yl)phenol	$C_{20}H_{22}O_4$

Fig. S3 Total Ion Current (TIC) chromatogram (left) and LMW phenolics produced (right) for *Phaeomoniella chlamydospora* metabolite extracts in negative (A) and positive (B) ionization mode. Peaks were selected by largest area and peak height.

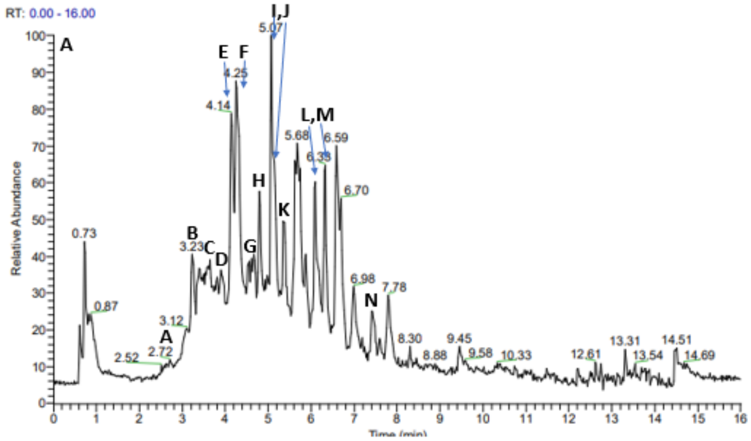

	Compound	Formula
A	D-pantothenic acid	$C_9H_{17}NO_5$
B	Citrazinc acid	$C_6H_5NO_4$
C	3-Coumaric acid	$C_9H_8O_3$
D	Lignicol	$C_{11}H_{12}O_6$
E	Phenylacetic acid	$C_8H_8O_2$
F	Caffeic acid[a]	$C_9H_8O_4$
G	Gallic acid[a]	$C_7H_6O_5$
H	Sinapic acid methyl ether[a]	$C_{12}H_{14}O_5$
I	Homogentisic acid[a]	$C_8H_8O_4$
J	(4S,5Z,6S)-4-(2-methoxy-2-oxoethyl)-5-[2-[(E)-3-phenylprop-2-enoyl]oxyethylidene]-6-[(2S,3R,4S,5S,6R)-3,4,5-trihydroxy-6-(hydroxymethyl)oxan-2-yl]oxy-4H-pyran-3-carboxylic acid	$C_{26}H_{30}O_{13}$
K	4-hydroxy-3-(3-methylbut-2-enyl)benzoic acid	$C_{12}H_{14}O_3$
L	2-Hydroxybenzothiazole	C_7H_5NOS
M	2-[2-[(Z)-pent-2-enyl]-3-[3,4,5-trihydroxy-6-(hydroxymethyl)oxan-2-yl]oxycyclopentyl]acetic acid	$C_{18}H_{30}O_8$
N	Nordentatin	$C_{19}H_{20}O_4$

[a]Known iron reducer

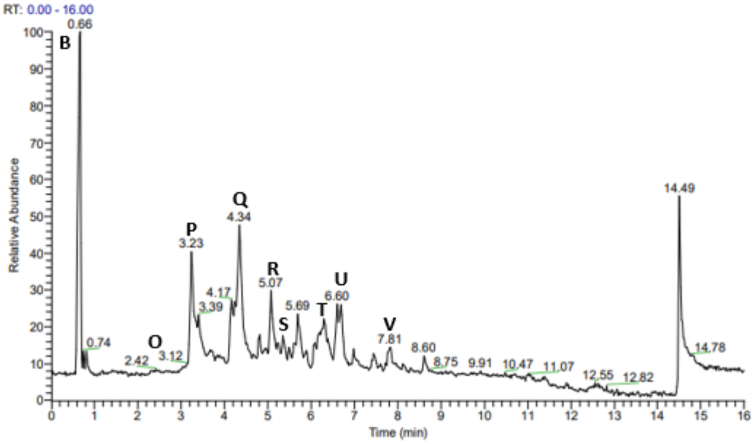

	Compound	Formula
O	N-Methyl-2-pyrrolidone	C_5H_9NO
P	Sorbic acid	$C_6H_8O_2$
Q	Sinapinic acid[a]	$C_{11}H_{12}O_5$
R	4-Methoxycinnamic acid	$C_{10}H_{10}O_3$
S	Tropine	$C_8H_{15}NO$
T	Benzothiazole	C_7H_5NS
U	L-Iditol	$C_6H_{14}O_6$
V	1-Dodecanoyllysolecithin	$C_{20}H_{42}NO_7P$

[a]Known iron reducer

Fig. S4 Total Ion Current (TIC) chromatogram (left) and LMW phenolics produced (right) for *Phaeoacremonium minimum* metabolite extracts in negative (A) and positive (B) ionization mode. Peaks were selected by largest area and peak height.
